# Mediterranean Diet Adherence Tools in Post-Treatment Breast Cancer Survivors: A Scoping Review of Measurement Approaches and Associated Outcomes

**DOI:** 10.3390/cancers18193185

**Published:** 2026-10-02

**Authors:** Katie E. Johnston, Aoife M. Twomey, Samantha J. Cushen

**Affiliations:** 1 Clinical Nutrition and Oncology Research Group, School of Food and Nutritional Sciences, University College Cork, T12 K8AF Cork, Ireland; kjohnston@ucc.ie; 2 Cancer Research @UCC, College of Medicine and Health, University College Cork, T12 K8AF Cork, Ireland; 3 School of Food and Nutritional Sciences, University College Cork, T12 K8AF Cork, Ireland; 118309093@umail.ucc.ie

**Keywords:** breast neoplasms, survivorship, mediterranean diet, review literature, anthropometry, nutritional status, quality of life

## Abstract

Background: Nutrition is an important yet underexplored determinant of cancer survivorship outcomes. Emerging evidence suggests that greater adherence to the Mediterranean diet (MedD) is associated with improved health status, enhanced quality of life (QoL), and reduced mortality. However, evidence specifically targeting post-treatment breast cancer survivors remains limited, and substantial heterogeneity in MedD scoring tools and adherence thresholds restricts comparability across studies. This review aimed to (i) identify and characterise the tools used to assess MedD adherence in breast cancer survivors following completion of active treatment, including their components, scoring systems, and adherence thresholds; and (ii) map the range of health outcomes that have been examined in relation to MedD adherence in this population. Methods: A scoping review was conducted in accordance with PRISMA-ScR and Joanna Briggs Institute guidelines. PubMed, Embase, Scopus, Web of Science, and CINAHL were searched (2000–2026) for English-language intervention and observational studies reporting MedD adherence in adult female breast cancer survivors after treatment completion. Two reviewers independently extracted data, which were synthesised descriptively, including study characteristics, MedD scoring tools, adherence outcomes, and clinical, metabolic, and QoL findings. The methodological structure protocol of this scoping review was retrospectively registered in the Open Science Framework. Results: Twenty studies (16 intervention, 4 observational; 16 non-overlapping cohorts; pooled n *N* = 4174, excluding non-cancer comparators) met inclusion criteria. Ten MedD scoring tools were identified, most frequently MEDAS-14 (original or modified, 7/20 studies), varying in components (range 9–18), scoring approach, and adherence thresholds. Most intervention studies reported improved MedD adherence following intervention, generally as a within-group change; between-group differences in adherence were reported less often and inconsistently significant. Anthropometric outcomes (body weight, body mass index (BMI), waist circumference) were most frequently improved, but significant differences between groups were found in only a small number of controlled studies. Mortality was assessed in only one observational cohort study, which found no association with breast-cancer-specific outcomes. Conclusions: This scoping review identified considerable heterogeneity in how MedD adherence is measured in breast cancer survivors, with ten different scoring tools identified across 20 studies. Although improvements in MedD adherence appear achievable, the evidence is limited by small sample sizes, heterogeneous assessment tools, and short intervention durations. Larger, standardised longitudinal studies are needed, particularly to determine whether MedD adherence influences cancer recurrence and survival, for which current evidence remains minimal.

## Simple Summary

Healthy eating is an important part of life after breast cancer treatment, yet the role of diet in long-term recovery is not fully understood. The Mediterranean diet includes plenty of vegetables, fruits, legumes, wholegrains, fish, and olive oil, and limits red and processed meats. It has been linked to better health and quality of life in many populations. This review looked at how adherence to the Mediterranean diet is measured in studies of women who have finished active cancer treatment for breast cancer, and whether following this diet is linked to better health outcomes. We found 20 studies using ten different tools to assess how closely women followed the Mediterranean diet. Most intervention studies showed that women improved their diet when supported to do so. In some studies, higher adherence was associated with healthier body weight, better blood sugar control, and some improvements in quality of life, though these findings come from small, short-term studies and do not show that the diet directly caused these changes. Only one study looked at whether the diet was linked to lower rates of cancer recurrence or to survival, and it found no clear benefit—so while other health improvements were seen, this does not mean the diet reduces cancer recurrence or improves survival. Results for cholesterol and inflammation were less consistent. Larger, longer-term studies are needed to confirm these benefits.

## 1. Introduction

According to the World Health Organisation (WHO), 2.3 million women were diagnosed with breast cancer in 2022, resulting in 670,000 deaths globally [1]. The international five-year breast cancer survival rate is above 80% [2] or 85% in Ireland, partly attributed to early detection [3] and advances in treatment [4]. Given that breast cancer survivors have complex and varied health needs throughout the different stages of their cancer journey, coordinated care is required to optimise health, health outcomes and co-morbidities post-cancer treatment [5]. Notably, a 2023 cohort study *(N* = 16,044) identified that post-treatment breast cancer survivors had a 70% higher risk of any cancer and a 45% higher risk of non-breast cancer, compared with the general population, after a median follow-up of 6.2 years [4].

Nutrition is one important, yet overlooked, aspect of multi-disciplinary care following the completion of active treatment [6]. Breast cancer survivors can be impacted by weight gain [7,8,9], a quicker bone mineral density decline [10,11], iatrogenic menopause [10,12,13], and decreased quality of life (QoL) [14] secondary to systemic anti-cancer treatment. Alterations to body composition, particularly adipose tissue mass, as a direct or indirect result of treatment, can worsen prognosis [7] and increase the risk of therapy-related morbidity and breast cancer-related mortality [9]. Current nutrition guidelines for breast cancer tend to be generic, with confusion around the role of nutrition in post-diagnosis survival and symptom management [7]. The European Society for Clinical Nutrition and Metabolism’s Clinical Nutrition in Cancer Practical Guideline recommends that cancer survivors maintain a healthy lifestyle, including being physically active and having a diet based on vegetables, fruits and wholegrains, and low in saturated fat, red meat and alcohol [15]. These recommendations align closely with World Cancer Research Fund (WCRF) Recommendations for Cancer Prevention [16] and follow similar principles to the Mediterranean diet (MedD). Furthermore, in 2020, the European Society of Menopause and Andropause endorsed the MedD for the management of menopause symptoms and long-term health [17], which is particularly relevant to this cohort. The MedD is a dietary pattern that includes high intakes of extra virgin olive oil, vegetables including leafy green vegetables, fruits, cereals, nuts and pulses/legumes, moderate intakes of fish and other meat, dairy products and red wine, and low intakes of eggs and sweets [18]. The MedD promotes unsaturated fats and fibre and limits saturated fat [19]. The MedD dietary pattern has evolved considerably since its initial characterisation in the 1960s through the Seven Countries Study [20] and formal representation in the 1995 Mediterranean Diet Pyramid [21]. Subsequent evidence from landmark studies in the late 1990s and early 2000s, including the Lyon Diet Heart Study [22] and the Greek EPIC cohort [23], reinforced its cardioprotective properties and highlighted regional transitions. The 2009 revision of the pyramid emphasised sustainability and cultural adaptability, reflecting its shift from a traditional regional diet to a globally applied model [24]. The MedD is thought to exert anti-inflammatory effects through its abundance of polyphenols and antioxidants [25], coupled with a low intake of pro-inflammatory foods such as processed products, sugary drinks, and red meat [26]; importantly, these benefits derive from the overall dietary pattern rather than individual components [27], with polyphenols in particular implicated in reducing inflammation and cancer recurrence [7]. The MedD has been shown to reduce and manage a range of conditions, including breast cancer [28], cardiovascular disease (CVD) [29], osteoporosis [10,11], diabetes [30], obesity [31], and metabolic syndrome [32], as well as to improve inflammatory profiles [7,33], thereby suggesting potential benefits for breast cancer survivors beyond current dietary guidelines for cancer prevention and cancer survivorship.

Adherence to MedD is commonly calculated using MedD scoring tools, of which there are several [34]. However, variation in these tools—including differences in scoring systems, component foods and adherence thresholds, leads to inconsistencies in interpretation and limits comparability across studies [34]. Even within Mediterranean countries, adaptations of these tools lack standardisation. [24]. Assessment of MedD adherence is uncommon in clinical practice, partly due to uncertainty around assessment and the complexity of some questionnaires [35]. However, MedD adherence scores may serve as useful indicators of dietary quality, allowing for clear assessment and modification of diet to help manage symptoms, and reduce the risk of chronic diseases and cancer recurrence.

To our knowledge, no existing review has systematically examined the methods used to assess MedD adherence among breast cancer survivors who have completed active treatment. Clarifying how adherence has been measured in this population, and what has been studied in relation to it, is a necessary first step toward standardising assessment and building comparable evidence.

This scoping review therefore aimed to (i) identify and characterise the tools used to assess MedD adherence in breast cancer survivors following completion of active treatment, including their components, scoring systems, and adherence thresholds; and (ii) map the range of clinical, metabolic, and QoL outcomes that have been examined in relation to MedD adherence in this population.

## 2. Methods

**Figure 1:**
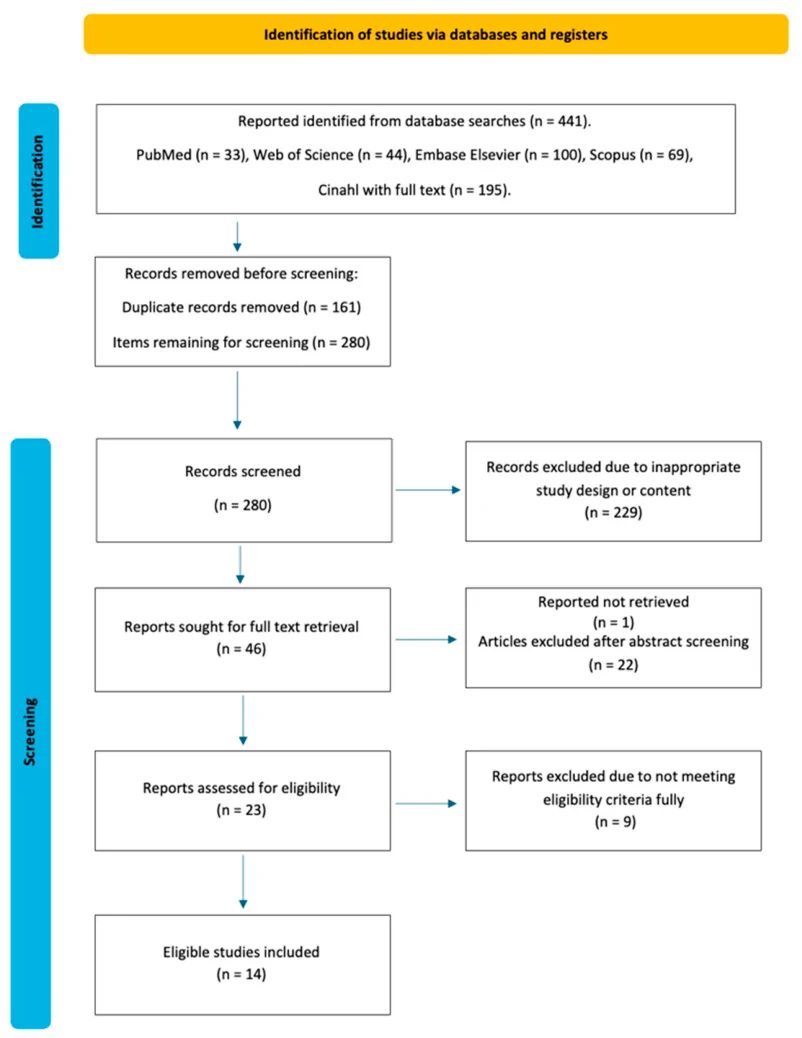
Preferred Reporting Items for Systematic Reviews and Meta-Analysis Flow Diagram.

This scoping review was conducted following Preferred Reporting Items for Systematic Reviews and Meta-Analyses guidelines for scoping reviews (PRISMA-ScR) [36] (Figure 1). The methodology of this scoping review was based on the Joanna Briggs Institute (JBI) methodology as reported by Kahlil et al. (2016) [37]. The review protocol was registered retrospectively on the Open Science Framework (OSF) (https://osf.io/cv3n2) [36].

### 2.1. Search Strategy and Information Sources

A systematic search was performed on 15 August 2026, across five electronic databases: PubMed, Embase Elsevier, Scopus, Web of Science and Cinahl, using Medical Subject Headings (MeSH), keywords and synonyms. A detailed search strategy and full search terms were developed with an academic librarian (Supplementary Table S1). The search strategy combined MeSH and relevant keywords related to the MedD, breast cancer, survivorship, and post-treatment populations, using Boolean operations (AND, OR) to structure the search. The time frame from 2000 to 2026 was selected because the MedD adherence tools in contemporary clinical and research use were all developed and validated from this point onwards, ensuring the search captured the most relevant and methodologically consistent research exploring MedD adherence in breast cancer survivors.

### 2.2. Eligibility Criteria

As scoping reviews aim to clarify key concepts and definitions in the literature and to identify the main characteristics and factors related to a concept [38], this review included studies that examined scoring tools used to assess adherence to the MedD among breast cancer survivors and their associated clinical outcomes. Studies were required to meet the predefined population, concept and context (PCC) framework as recommended by the JBI methodology framework [37]. Only peer-reviewed studies published in English between January 2000 and August 2026 were included to ensure quality and accessibility. Inclusion criteria specified that the population consisted of adult female breast cancer survivors who had completed primary treatment, defined as finishing primary curative therapies, including surgery, chemotherapy, and/or radiotherapy. Participants receiving ongoing adjuvant endocrine therapy (e.g., tamoxifen, aromatase inhibitors) were eligible for inclusion, as endocrine therapy represents long-term maintenance treatment rather than active cytotoxic or surgical treatment; endocrine therapy status could not be consistently verified or excluded at the level of individual primary studies, as it was inconsistently reported by primary study authors. Participants were thus considered post-active treatment once they had completed these primary curative interventions (surgery, chemotherapy, and/or radiotherapy) and entered the survivorship or follow-up phase. Ongoing endocrine therapy was not considered active primary treatment for the purposes of this definition, given that endocrine therapy is commonly prescribed as long-term risk reduction adjuvant therapy during survivorship. Where studies used different definitions of “post-treatment” or survivorship, eligibility was assessed against this predefined operational definition based on the treatment status reported by the study authors. Studies must have examined MedD adherence either through direct dietary intervention or observational assessment using MedD scoring tools. Both experimental (randomised controlled trials (RCTs) and observational (cohort, case–control, cross-sectional) study designs were eligible to capture the full breadth of available evidence on MedD adherence tools and their outcomes. No restrictions were imposed on comparator groups, allowing inclusion of studies with varying dietary or non-dietary comparisons. Gray literature, such as unpublished theses, dissertations, and conference abstracts, was excluded due to resource constraints. Consistent with methodological guidance for scoping reviews, formal quality appraisal or risk of bias assessment was not undertaken [37].

### 2.3. Data Charting and Synthesis

A standardised, pilot-tested data extraction form was used on five relevant full text articles, before full data extraction with two reviewers independently charting data for each study. Disagreements were resolved by consensus with a third reviewer. Following guidance from the JBI methodology for Scoping Reviews [37], extracted data included study characteristics (author, year, country, design), participant characteristics (age, body mass index (BMI), treatment details, time since treatment completion), MedD adherence scores and scoring tools used, primary outcomes related to diet adherence, and key findings relevant to the breast cancer survivor population. A descriptive synthesis was independently conducted by all authors to summarise and map the evidence according to key themes, including study characteristics, MedD adherence scoring approaches and tools, and primary outcomes. Results are presented descriptively in tables, with simple quantitative analyses such as percentages and frequencies calculated using Excel. Any *p*-values reported in the included studies are also summarised descriptively (*p* < 0.05 or *p* < 0.001) to provide additional context regarding study findings.

## 3. Results

### 3.1. Study Selection

As presented in Figure 1, a total of 566 articles were identified through database searches. After the removal of 222 duplicates, 344 articles remained for title and abstract screening. Following this, full-text reviews were conducted on 27 studies to assess eligibility based on the predefined inclusion criteria. Twenty studies met the inclusion criteria, representing a total of 4174 post-treatment breast cancer survivors.

### 3.2. Summary of Included Studies

The twenty included studies were published between 2011 and 2026, comprising twelve RCTs (60%), four single-arm intervention studies (20%), one cross-sectional observational study (5%), and three cohort studies (15%) (Table 1). Two clusters of studies analysed overlapping participant cohorts. Donati Zeppa et al. (2023) [39], Natalucci et al. (2023) [40], and Annibalini et al. (2026) [41] reported on the same *MoviS Trial* (NCT04818359)*;* Donati Zeppa et al. examined a subgroup (*n* = 20) of Natalucci et al.’s full cohort (*n* = 30) while Annibalini et al. subsequently reported a larger biomarker subgroup (*n* = 50) which included a control arm which was added once COVID-19-related restrictions eased. Similarly, Cho et al. (2020) [42], Kwon et al. (2020) [43], and Choi et al. (2026) [44] reported on the same Korean pilot trial (NCT03581630). Sample sizes across the 20 publications ranged from 16 [43] to 2729 [45], with a median of 56.5 (IQR 36.5–118.5); the pooled total across the 16 non-overlapping study cohorts, excluding non-cancer comparators, was *n* = 4174. Across the included studies, participants were recruited at various points in the cancer care continuum, from immediate post-treatment survivorship to long-term survivorship, with follow-up lasting several decades. None of the included studies stratified analyses by tumour subtype. All required participants had to have completed active treatment (chemotherapy, radiotherapy, surgery), with variability in completion requirements of adjuvant endocrine therapy; only 8 of 20 studies reported the actual proportion of participants receiving endocrine therapy (Table 1).

### 3.3. Tools Used to Assess Mediterranean Diet Adherence

#### 3.3.1. Tools Identified and Their Components

Several versions of MedD adherence scores exist, each operationalising dietary recommendations slightly differently. Ten different MedD scoring tools were used across the 20 studies (Table 2). The Mediterranean Diet Adherence Screener (*MEDAS*), including its modified and culturally adapted variants (e.g., K-MEDAS, Modified MEDAS 13-/14-item) was the most commonly used scoring tool, used in 35% (*n* = 7) of included studies. Other studies included a modified version of the Alternate Mediterranean Diet Score (*aMED*) (*n* = 1) [46], *MEDI-LITE* (*n* = 1) [45], the Mediterranean Diet Score (*MDS*) tool (*n* = 1) [47], *MedDiet* (*n* = 1) [48], a Cardioprotective Mediterranean Diet Score (*n* = 1) [46], the Italian Mediterranean Diet Index (*n* = 2), the 17-item PREDIMED-Plus questionnaire (*n* = 1) [49], and the QueMD questionnaire (*n* = 1) [50]. The number of components included in the MedD scoring tools ranged from 9 to 18 (Supplementary Table S2).

#### 3.3.2. Scoring Systems and Adherence Thresholds

Overall, the scoring tools consistently encourage vegetables, fruits, legumes, fish and seafood, olive oil, nuts and moderate alcohol, whilst consistently discouraging red and processed meats, butter, cream and sugary foods and drinks (Supplementary Table S2). However, the tools differ in how adherence is scored, and this variation follows two separate categories: how thresholds are defined, and who the tool was designed for. In terms of threshold definition, some tools use binary scoring (a point either awarded or not), others use portion-based cut-offs (an absolute, component-level quantity, such as two or more servings of vegetables per day), and others use median- or tertile-based scoring, where adherence is defined relative to the intake distribution of the source population rather than against a fixed quantity. In terms of population or purpose, several tools have been adapted for specific populations or purposes, including Italian cohorts, US cohorts and intervention weight-loss studies. The *MEDAS 14* tool uses clear portion-based cut-offs (e.g., two servings/day vegetables) and emphasises olive oil, legumes, fish, nuts and moderate wine [51]. The *aMED* tool adapts this approach for North American populations, using median intakes as the threshold for foods such as fruits, vegetables, legumes, wholegrains and fish [45]. The original MDS (OMDS) uses simple binary scoring across 9 items, with adherence for each component defined relative to the sex-specific median intake of the study population [23]. The *MDS* variant uses a considerably larger, 0–5 point scale across a wider set of components, rewarding consumption of plant foods and white meat while discouraging red meat [52]. The *MEDI-LITE* tool uses a graded 0–2 point system based on frequency of consumption for a broader set of food groups [53]. The *Italian Mediterranean Index* tool is tailored to Italian eating habits, highlighting pasta, tomato-based sauces and olive oil, while limiting meat and dairy [54]. A separate Modified MEDAS 13-item tool, developed for a Korean cohort, retains the original MEDAS framework but removes the alcohol item entirely, using a lower 13-point maximum and a ≥9-point threshold to define high adherence [50]. The QueMD tool, validated in an Italian cancer-screening population, uses a graded 0–2 point frequency-based scoring system across 15 food items to derive an aMED-equivalent score, giving it a substantially wider achievable range than the other tools in this review [50]. Finally, the *MedDiet* tool designed for weight-loss trials, adapted *MEDAS* with stricter criteria on sugary drinks, refined grains and extra virgin olive oil [51]. Because of this variation, maximum achievable scores ranged from 9 to 18 across tools (Table 2). More importantly, the point at which overall adherence was classified as “high” was defined inconsistently between studies and, in several cases, was not reported as a discrete cut-off at all, meaning that “high adherence” findings are not necessarily comparable across the studies included in this review.

### 3.4. Changes in MedD Adherence

Of the 16 studies that compared pre- and post-intervention adherence, 4 publications represented repeated adherence assessments of two previously counted intervention cohorts. Correcting for this overlap, 12 unique intervention cohorts provided independent pre-/post-intervention adherence data, of which 9 reported statistically significant improvements.

Ruiz-Vozmediano et al. (2020), using the *MEDAS 14* excluding alcohol, found that MedD adherence increased significantly following the MedD intervention; 44% reported good adherence at baseline to 73% post intervention (*p* < 0.05) compared to the non-significant decrease in the control arm (31% to 22%) (*p* < 0.001) [55]. Acito et al. (2023) [46] reported an increased adherence score over the period of the COVID19 lockdown: 8.20 ± 1.88 to 9.92 ± 2.27 (*p* < 0.05). Post-COVID-19 pandemic, 65.2% were classified as high adherence, compared to 25.4% at baseline [46]. The mean adherence to the *MEDAS 14* score was 9.1 ± 2.1 [56]. Furthermore, there was a significant increase in intakes of MedD-recommended foods and an increase in the MedD adherence score from 10.73 ± 2.92 to 11.82 ± 3.46 (*p* < 0.05). White et al. (2025), using the MEDAS 14 tool over a 12-week intervention, also reported a statistically significant increase in MedD adherence (6.7 to 7.9, *p* < 0.001), alongside significant improvements in BMI and menopausal symptom scores [57].

Pellegrini et al. (2020) [58] found no statistically significant change in MedD adherence following a 4-month intervention trial in both the MedD alone and the MedD with probiotics. Using the *MEDI-LITE* score, Magro et al. (2019) reported statistically significant improvements in adherence scores in both groups over the intervention period (*G1: 13.5 ± 1.3 to 14.6 ± 1.0* [*p* < 0.05], *G2: 12 ± 2.7 to 13.2 ± 2.2* [*p* < 0.05]), but no statistically significant differences were reported between groups [47]. Following an 8-week intervention, Kwon et al. (2020) reported a statistically significant improvement in MedD adherence score, increasing from 7.8 ± 1.9 at baseline to 10.7 ± 1.51 at endpoint (*p* < 0.001) [43]. Using the MoviS cohort, Natalucci et al. (2023) reported a statistically significant 28% increase in MedD adherence (*p* < 0.001) over the 3-month intervention period [40] and Donati Zeppa et al. (2023) [39] reported a statistically significant increase in MedD adherence (*7.65 ± 2.32 to 9.25 ± 2.38, p* < 0.05). A subsequent biomarker subgroup analysis of the same MoviS trial, Annibalini et al. (2026), reported that MedD adherence increased in both study arms [41]. Braakhuis et al. (2017), using the *MEDAS 14* tool, also reported a statistically significant increase in pre- and post-intervention scores: 6.35 ± 2.81 vs. 9.80 ± 1.52 (*p* < 0.05) [7].

**Table 1:** Summary of characteristics and results of included studies (*n* = 20), presented in chronological order of publication.

#	Study	Study Characteristics	Time Since Surgery/Chemo/RT; Endocrine Therapy	MedD Adherence Tool	Objective	Results
1	Kim [45] (2011)	Cohort Study *Sample*: 2729 breast cancer survivors *Period:* Diagnosis between 1978 and 1998; follow-up until 2004 *Place:* United States	Time since surgery/chemo/RT: Not reported. The diet was assessed ≥12 months post-diagnosis (not specifically post-surgery/treatment); women with diet assessed during active treatment were excluded. Endocrine therapy: Tamoxifen use ranging ~56–67%.	aMed	Breast cancer-specific mortality, total mortality, and recurrence risk.	No clear impact observed on breast cancer recurrence or overall breast cancer mortality. Higher adherence to MedD and other diet quality indices was associated with lower non-breast cancer mortality, especially cardiovascular deaths
2	Finocchiaro [59] (2016)	Single-Arm Intervention Study *Sample:* 100 breast cancer survivors *Period:* between 2009 and 2010 *Place*: Italy	Surgery ≤ 12 months; chemo completed ≥6 months prior (thresholds, not means). Endocrine therapy 76% at baseline.	Italian Mediterranean Index	MedD adherence, reducing BMI and WC, and promoting physical activity in breast cancer survivors.	Significant decrease in BMI and WC observed at 2- and 6-month post-intervention. Adherence to the MedD improved significantly Physical activity increased
3	Braakhuis [7] (2017)	Randomised Controlled Trial *Sample:* 50 breast cancer survivors *Period:* 12-week intervention in 2016 *Place:* New Zealand	3 months–3 years post-active treatment (surgery/chemo); RT timing NR. Endocrine proportion NR.	MEDAS 14	MedD adherence, changes in QoL, weight, BMI, cardiovascular and metabolic biomarkers in breast cancer survivors	Improved adherence to MedD after nutrition education intervention Significant decrease in BMI and WC in the MedD group compared to controls
4	Zuniga [60] (2019)	Randomised Controlled Trial *Sample:* 125 breast cancer survivors *Period:* 6 months in 2016 *Place:* United States	Combined threshold: completed treatment ≥ 2 months prior. Majority > 24 months since diagnosis. Endocrine proportion NR.	Cardioprotective Mediterranean Diet Score	MedD adherence, changes to inflammatory biomarkers and health outcomes	Improved adherence to MedD in the intervention group Significant reduction in calorie intake, in the intervention group
5	Magro [47] (2019)	Randomised Controlled Trial *Sample:* 21 breast cancer survivors *Period:* 2-month intervention in 2018 *Place:* Italy	NR—eligibility only requires ‘previous breast cancer’/‘complete remission’. Endocrine proportion NR.	MEDI-LITE	MedD adherence, WCRF cancer prevention adherence, changes to body weight, BMI, WC and HC physical activity, lower body strength	Improved adherence to MedD BMI reduction, weight reduction Improvements in waist and hip circumference Increased weekly physical activity by 35 min
6	Cho [42] (2020) ^b^	Randomised Controlled Trial *Sample*: 44 breast cancer survivors (Group A *n* = 21 diet-alone, Group B *n* = 23 diet + drug) Parent trial total n = 72, including 28 non-cancer comparators (Group C) * Period: 8-week intervention conducted between 2017 and 2018 *Place: South Korea*	NR—requires treatment (surgery/chemo/RT/hormonal) to be ‘completed’, no time threshold. Active tamoxifen was an exclusion. Endocrine proportion NR.	Modified MEDAS 13-item	MedD adherence, body weight, metabolic biomarkers, nutrient intake, QoL	Improved adherence to MedD Significant BMI reduction, weight reduction Significant improvements in fasting glucose, insulin and insulin resistance Improvements in QoL
7	Ruiz-Vozmediano [55] (2020)	Randomised Controlled Trial *Sample*: 63 breast cancer survivors *Period:* 6-month intervention in 2016 *Place:* Spain	>12 months post-treatment (combined threshold). Endocrine therapy: control 84.9%, intervention 88.2%.	MEDAS 14	MedD adherence, QoL, clinical measurements	Improved adherence to MedD Improvement in BMI and body weight Significant improvements in clinical measurements (total cholesterol, triglycerides) Significant improvements in specific QoL domains (physical, social and role functioning)
8	Kwon [43] (2020) ^b^	Single-Arm Intervention Study *Sample:* 16 breast cancer survivors *Period:* 8-week intervention *Place:* South Korea	≥5 years post-treatment (combined threshold, any modality). Endocrine proportion NR.	K-MEDAS 14 ^	MedD adherence, Metabolic and anthropometric measurements (BMI, WC, fasting glucose, fasting insulin), extracellular vesicle microRNA (miRNA) expression profiles	Improved adherence to MedD Significant improvement in BMI, WC, fasting glucose, fasting insulin, and insulin resistance after 8-week intervention Extracellular vesicle miRNAs significantly altered after intervention
9	Pellegrini [58] (2020)	Randomised Controlled Trial *Sample:* 26 breast cancer survivors *Period:* 4-month intervention conducted between 2017 and 2018 *Place:* Italy	NR—eligibility requires ‘cancer-free’ only. Endocrine proportion NR.	Italian Mediterranean Index	MedD adherence, changes in gut microbiota composition, changes in BMI, body weight, WC and metabolic parameters	No significant improvement in adherence to MedD Significant improvements in anthropometric and metabolic parameters (including weight, BMI, WC, waist/hip ratio, insulin, fasting glucose)
10	Negrati [48] (2021)	Cohort Study *Sample:* 80 breast cancer survivors *Period:* Recruitment from 2012 to 2014 *Place:* Italy	No pharmacological/RT treatment for ≥2 months prior to enrolment (combined). Endocrine proportion NR.	Mediterranean Diet Score (MDS)	MedD adherence, changes in anthropometric parameters, metabolic parameters	MDS was positively correlated with blood β-carotene and lycopene and negatively with retinol No significant correlations were found between MDS and cardiometabolic parameters (BMI, glucose, insulin, HOMA-IR, lipids) in this cohort
11	Alvarez-Bustos [56] (2021)	Cross Sectional Observational Study *Sample:* 180 breast cancer survivors *Period:* Recruitment from 2020 to 2021 *Place:* Spain	Mean 856 days (~28 months) since diagnosis. Chemo 73%, RT 65%, Endocrine therapy 81% (AI 25%).	MEDAS 14	MedD adherence Fatigue, QoL, clinical and anthropometric data	Tumour characteristics, prior treatments, physical activity, cardiorespiratory fitness, and MedD adherence showed no consistent association with fatigue severity
12	Natalucci [40] (2023) ^a^	Randomised Controlled Trial *Sample:* 30 breast cancer survivors *Period:* 3-month intervention, 2020–2021 *Place:* Italy	≤12 months since surgery; ≥6 months since completion of RT/chemo. Endocrine therapy: 80% on current therapy (26.7% tamoxifen, 53.3% AI, 20% none)	MeDiet	MedD adherence, anthropometrics, cardiorespiratory fitness, physical activity level, and cardiometabolic/hormonal biomarkers	Improved MedD adherence and cardiorespiratory fitness sustained to 24 months; BMI and waist circumference improved short-term only; glycaemic control and insulin resistance improved up to 12 months but not sustained at 24
13	Acito [46] (2023)	Cohort Study *Sample:* 224 breast cancer survivors enrolled in the Diana Web study *Period:* Evaluated data before, during, and after the first two waves of the COVID-19 pandemic (2019–2021) *Place:* Italy	Majority ≥ 5 years, ~1/3 > 10 years since diagnosis. Endocrine proportion NR.	PREDIMED PLUS Energy Restriction Questionnaire ~	MedD adherence, adherence to WCRF recommendations, changes in physical activity	Compared to baseline, there was a significant increase in adherence to MedD in the Diana Web cohort. No significant changes were found in MedD or WCRF adherence during and after the lockdown, indicating durability of dietary habits
14	Garcia-Unciti [61] (2023)	Randomised Controlled Trial Sample: 43 postmenopausal breast cancer survivors. Randomised 1:1 to Control Group (CG, *n* = 22) vs. Intervention/Exercise Group (IG, *n* = 21) *Period:* January 2012–September 2017. Individual follow-up per participant: 1 year (baseline, 6-month, 12-month assessments) *Place:* Spain	Enrolled at the point of starting AI treatment. Inclusion criteria states transition from active treatment (chemo/RT/surgery) to AI initiation.	Original Mediterranean Diet Score (OMDS)—Trichopoulou et al., 2003 [23] (validated in Spanish population)	Impact of combined training program (twice weekly) on body composition and MedD adherence	Decrease in weight in intervention group 19–21% of the total sample had **high** adherence to MedD high-adherence proportion slightly declined over the year (20.8% to 18.6%)
15	Donati Zeppa [39] (2023) ^a^	Single-Arm Intervention Study *Sample:* 20 breast cancer survivors *Period:* 12-week intervention, 2020–2021. *Place:* Italy	Mean 10.2 ± 3.1 months since diagnosis. Endocrine therapy 75% (tamoxifen 25%, AI 50%).	MeDiet (MEDAS 14)	MedD adherence, changes in gut microbiota	Significant beneficial changes in gut microbiota composition; positive correlations between MedD adherence and beneficial gut bacteria
16	White [57] (2025)	Single-Arm Intervention Study *Sample:* 72 enrolled *Period:* 12-week intervention *Place:* United Kingdom	NR—not broken down by modality; Endocrine status included in eligibility only.	MEDAS 14	MedD adherence, BMI, menopausal symptoms, fatigue in breast cancer survivors.	Improved MedD adherence (6.7 to 7.9, *p* < 0.001) Significant BMI reduction (26.9 to 26.3 kg/m^2^, *p* < 0.001) and reduced menopausal symptom score (*p* < 0.001); fatigue unchanged (*p* = 0.37)
17	Ruiz-Molina [49] (2025)	Randomised Controlled Trial (open-label, 2 arms) *Sample*: 112 randomised; 94 completed (intervention *n* = 43, control *n* = 51) *Period:* 6 months (12-week intensive phase) *Place:* Spain	≥6 months post-chemo/RT (eligibility threshold). Endocrine proportion NR.	PREDIMED-Plus (17-item) +	MedD-based lifestyle intervention effects on lymphedema, body composition, inflammatory markers in overweight breast cancer survivors.	Intervention group showed significantly greater improvements in BMI, waist circumference, fat mass, and quadriceps strength ≥5% weight loss associated with greater reduction in affected-limb volume; no significant change in circulating cytokines
18	Annibalini (2026) ^a^ [41]	Randomised Controlled Trial (MoviS trial subgroup) *Sample:* 50 (control *n* = 26, intervention *n* = 24) *Period:* 3-month intervention *Place*: Italy	≤12 months post-surgery, ≥6 months post-chemo/RT Control group (*n* = 26): Tamoxifen 2 + Aromatase Inhibitor 14 = 16 on endocrine therapy Intervention group (*n* = 24): Tamoxifen 3 + Aromatase Inhibitor 11 = 14 on Endocrine therapy	Modified MeDiet 14-item	Effects of the MoviS lifestyle intervention on IGF-1, IGFBP1 and IGFBP3 in breast cancer survivors.	IGFBP1 increased and IGFBP3 decreased in both groups; IGF-1 response depended on baseline level (U-shaped relationship with fitness/fat-mass change) MedD adherence improved more in the intervention group (6.8 to 8.2) than control (7.1 to 7.3)
19	Choi [44] (2026) ^b^ *	Randomised Controlled Trial (Groups A/B randomised BC survivors; Group C non-randomised non-cancer comparator) *Sample:* 44 breast cancer survivors (Group A *n* = 21 diet-alone, Group B *n* = 23 diet + drug) Parent trial total *n* = 72, including 28 non-cancer comparators (Group C) * Period: 8-week intervention *Place:* South Korea	Inclusion criteria required completion of surgery/chemo/RT/hormonal therapy. Anyone currently taking tamoxifen specifically was excluded. % on Endocrine therapy NR	Modified MEDAS 13-item	Circulating polyamines as candidate metabolic-response markers to MedD ± naltrexone/bupropion in breast cancer survivors.	High-MedD-adherence proportion increased in both BC-survivor groups (Group A 40.0% to 90.0%; Group B 42.9% to 92.9%) MedD-related metabolic improvements accompanied by changes in circulating polyamines; spermine increased only in the diet-alone group
20	Gnagnarella [50] (2026)	Randomised Controlled Trial (InForma Study) *Sample:* 255 breast cancer survivors *Period:* 6-month intervention, followed up for 18 months *Place:* Italy	>6 months post-treatment (eligibility threshold). % on Endocrine therapy: 85.9% (219/255)	15-item QueMD questionnaire	Dietary-pattern change (Mediterranean/Sweet/Western) over time and predictors of >5% weight loss in overweight/obese breast cancer survivors.	MedD adherence improved across the whole cohort regardless of study arm; consumption on Sweet/Western patterns declined over follow-up Diet-only and diet + physical-activity arms (vs. minimal intervention), and age ≥ 60y, predicted > 5% weight loss

**Abbreviations:** AI, Aromatase Inhibitor; BMI, Body Mass Index; HC, Hip Circumference; HOMA-IR, Homeostatic Model Assessment for Insulin Resistance; MedD, Mediterranean Diet; NR, Not Reported; QoL, Quality of Life; RT, Radiotherapy; WC, Waist Circumference; WCRF, World Cancer Research Fund. **^** validated culturally adapted 14-item Mediterranean Diet Adherence Screener (K-MEDAS). ~ PREDIMED PLUS Energy Restriction Questionnaire is a Mediterranean diet assessment tool tailored specifically for the Diana Web platform and population context with slight differences in food components scored. + 17-item Mediterranean Diet (MedDiet) questionnaire is an extended version of the MEDAS 14-item screener, used in the PREDIMED-Plus trial, designed to assess adherence to an energy-restricted Mediterranean diet. **^a^** Studies 12, 15 and 18 report overlapping participants from the same trial (MoviS, NCT04818359). To avoid double-counting, MoviS-trial participants are counted once in the pooled total, using the largest reported *n* (*n* = 50, Annibalini et al. 2026, [41]). **^b^** Studies 6, 8 and 19 report overlapping participants from the same trial (NCT03581630, South Korea). To avoid double-counting, this trial’s participants are counted once in the pooled total, using the breast-cancer-survivor total reported by Choi et al. (2026) (*n* = 44) [44]. * The Cho/Choi trials (NCT03581630) also enrolled a non-cancer comparator group (Group C, *n* = 28) receiving MedD plus naltrexone/bupropion [42,44]. These participants are not breast cancer survivors and are excluded from all breast-cancer-survivor participant totals reported in this review. Pooled unique breast-cancer-survivor total: *n* = 4174, derived from 20 publications representing 16 unique study cohorts, after collapsing overlapping-cohort clusters (a, b) and excluding non-cancer comparators (*). Endocrine therapy in this table refers to tamoxifen, aromatase inhibitors (e.g., anastrozole, letrozole, exemestane), and related hormonal agents used to suppress oestrogen activity in hormone receptor-positive breast cancer.

**Table 2:** Overview of Mediterranean Diet Adherence Scoring Tools Used in Included Studies.

Scoring Tool (Author, Year)	Scoring Approach	How Adherence Was Defined	Studies Using Tool, n (of 20)
**Original Mediterranean Diet Score** (OMDS) (Trichopoulou et al., 2003) [23]	Binary (point awarded per criterion), 9 items, 0–9 scale	Sex-specific median of study population as cutoff	1
**Cardioprotective MedD Score** (Martínez-González et al., 2004) [62]	Binary (point awarded per criterion)	Portion-based cut-offs	1
**MEDAS 14** (Schröder et al., 2011) [51]	Binary (point awarded per criterion)	Portion-based cut-offs	7 *
**aMED** (Kim et al., 2011) [45]	Binary (point awarded per criterion)	Median intake of source population	1
**Mediterranean Diet Score (MDS)** (Demetriou et al., 2012) [52]	Graded (0–5 points per criterion, reverse-scored for red meat/poultry/dairy), 0–55 scale	Frequency-of-consumption cut-offs (fixed categories, not population median)	1
**MEDI-LITE** (Sofi et al., 2013) [53]	Graded (0–2 points per criterion), 0–18 scale	Portion-based cut-offs derived from pooled literature data (weighted mean ± 2 SD across cohort studies)	1
**Italian Mediterranean Index** (Agnoli et al., 2013) [54]	Binary (11 items: 6 scored for high intake, 4 for low intake, 1 for alcohol)	Tertile of source population distribution	2 ^
**Modified MEDAS 13-item** (Cho et al., 2020) [42]	Binary, wine/alcohol item removed, 0–13 scale	Portion-based cut-offs	3 †
**PREDIMED-PLUS** (Martínez-González et al., 2019) [63]	Binary, 17 items (stricter criteria: extra-virgin olive oil only, refined grains, sugary drinks)	Portion-based cut-offs	2 ~
**QueMD** [50]	Binary (point awarded per criterion), 0–9 scale	Portion- and frequency-based cut-offs aligned with dietary recommendations	1

The number of individual dietary components per tool ranged from 9 to 18. Full component-by-component scoring criteria and operationalisation of recommendations for each tool are provided in Supplementary Table S1. * The following studies included in this scoping review used the MEDAS 14 scoring tool to assess MedD adherence: Alvarez-Bustos et al., 2021; Braakhuis et al., 2017; Natalucci et al., 2023; Ruiz-Vozmediano et al., 2020; Donati Zeppa et al., 2023; White et al., 2025; and Annibalini et al., 2026 [7,39,40,41,55,56,57]. ^ The following studies included in this scoping review used the Italian Mediterranean Index to assess MedD adherence: Pellegrini et al., 2020; Finocchiaro et al., 2016 [58,59]. † Cho et al., 2020 (parent trial); Kwon et al., 2020 (nested sub-study of Group A participants from the same trial); and Choi et al., 2026 (secondary/exploratory analysis from the same trial) all applied the same modified 13-item MEDAS instrument (alcohol item removed, 0–13 scale) [42,43,44]. Kwon et al. (2020) used a Korean-adapted version (K-MEDAS) [43]. ~ The following studies included in this scoping review used the PREDIMED-PLUS: Ruiz-Molina et al. 2025 and Acito et al. 2023 [46,49].

Finocchiaro et al. (2016) [59] reported significant improvements in intakes of individual MedD components, including vegetable (*p* < 0.001), fruit (*p <* 0.05), pulses (*p* < 0.001), olive oil (*p* < 0.001) and fish (*p* < 0.001). Furthermore, authors highlighted the significant decrease in non-typical Mediterranean foods, including red meat (*p* < 0.001), potatoes (*p* < 0.05) and alcohol (*p* < 0.05). Cho et al. (2020) reported on macro and micronutrient level changes, reporting that dietary fibre intake and the ratio of monounsaturated fatty acids to saturated fatty acids increased (*p* < 0.001 and *p* < 0.05, *respectively*) [42]. A subsequent secondary analysis of the same trial, Choi et al. (2026), reported an increase in the proportion of participants classified as high-adherence rather than a mean adherence score: overall 37.5% to 85.7%, with similar increases in both breast-cancer-survivor arms (Group A, diet-alone: 40.0% to 90.0%; Group B, diet + drug: 42.9% to 92.9%) and the non-cancer comparator group (Group C: 31.8% to 77.3%) [44]. Similarly, Alvarez-Bustos et al. (2021) reported on adherence to individual components, finding the lowest adherence was to wine (19%) and pulses (22%) whilst the highest adherence was to olive oil as the main fat (99%) and white meat over red meat (88%) [56]. Zuniga et al. (2019) also reported on changes to individual components following a 6-month intervention between arms, with the overall score significantly increasing between intervention and control (+1.6 vs. + 0.2, *p* < 0.001) [60]. Adherence to recommendations for red meat (64% to 86.4%*, p* < 0.05), fish (13.6% to 35.6%*, p* < 0.05) and confectionary (69.5% to 86.4%*, p* < 0.05) increased [60].

Gnagnarella et al. (2026), in a secondary analysis of the InForma trial using a 9-item QueMD-derived subscore, reported that MedD adherence improved across the full 24-month follow-up period regardless of study-arm assignment; specific pre- and post-intervention adherence values were not reported in the accessible extract [50].

### 3.5. Clinical Outcomes Associated with Mediterranean Diet Adherence

As noted in Section 2.1, no formal risk of bias or quality appraisal was undertaken, consistent with scoping review methodology. The outcomes below are therefore reported descriptively, mapping what was measured, how, and in what study design, rather than synthesising intervention effectiveness. Reported changes reflect within-group or between-group differences as originally presented by each study, and the study design is reported alongside each finding to aid interpretation. The frequency of statistically significant findings should not be interpreted as evidence of intervention effectiveness.

Clinical outcomes examined across the 20 included studies span five broad domains: mortality/recurrence (1 study), anthropometric measures (16 studies), blood lipid biomarkers (11 studies), glycaemic control (12 studies), inflammatory markers (5 studies), and quality of life (4 studies). Measurement methods, study design, and the direction of reported change for each study and outcome domain are summarised in Table 3.

Mortality and recurrence were assessed only by Kim et al. (2011) [45], in a prospective cohort of 2729 post-treatment breast cancer survivors; higher Mediterranean diet adherence was not associated with reduced total mortality, breast-cancer-specific mortality, or recurrence overall, though a significant association with lower non-breast-cancer mortality was reported among women with low physical activity levels.

## 4. Discussion

This scoping review characterised the tools used to assess MedD adherence in breast cancer survivors following completion of active treatment, identifying 20 studies\across 16 unique cohorts and ten distinct scoring tools. Beyond the two overlapping cohorts identified within this total count, the evidence base is also concentrated in a small number of research groups. For example, 8 of the 20 studies came from Italian research groups, and some involved the same authors. For instance, Finocchiaro et al. [59] and Pellegrini et al. [58] shared three co-authors. This concentration of studies within a limited number of research groups may limit the extent to which the findings have been independently replicated.

Assessment of MedD adherence in this population was heterogenous; no single tool dominated, and adherence thresholds differed across studies. The MEDAS 14 tool was the most commonly used, accounting for 7 of 20 (35%) of included studies [7,39,40,42,43,55,56]. Despite sharing a common core, promoting vegetables, fruit, legumes, fish and olive oil while discouraging red and processed meat, the tools differed in ways that limited direct comparison of “adherence” across studies. Some tools (MEDAS-14, the Cardioprotective MedD Score, PREDIMED-PLUS) apply fixed, portion-based cut-offs, meaning that the same intake receives the same score regardless of the population being assessed. In contrast, others (aMED, OMDS, and the Italian Mediterranean Index) define adherence relative to the median or tertiles of intake within the study population. Under the latter approach, a participant could be classified a “high adherer” in one cohort and a “low adherer” in another despite reporting identical intake, limiting the comparability of adherence estimates across studies. Tools also varied in the number and type of dietary components assessed (9–18 items), with inconsistent inclusion of alcohol, dairy, and wholegrains, and limited justification or validation of adherence thresholds.

This is unsurprising, given the origins of the MedD measurement. Most tools identified in this review, including MEDAS-14 and the Cardioprotective MedD Score, were originally developed for cardiovascular disease prevention rather than oncology, reflecting the field’s historical roots in studies such as the Seven Countries Study and the Lyon Diet Heart Study [20,22]. Their subsequent use in cancer and survivorship populations therefore represents a repurposing of existing measures rather than the use of tools specifically developed for these populations. Of particular relevance to cancer survivorship, most tools score moderate alcohol intake favourably, reflecting their cardiovascular origins, despite WCRF guidance recommending alcohol avoidance; alcohol-excluded tools, such as the modified 13-item MEDAS used by Choi et al. (2026) [44], address this discrepancy. This issue is illustrated by Álvarez-Bustos et al. (2021) [56], who used the standard 14-item PREDIMED and reported wine as the dietary component with the lowest adherence in their cohort, with only 19% of participants meeting the scoring criterion. Consequently, 81% of participants received no points for this component. In a cancer population, where avoiding alcohol is recommended, this raises the possibility that a cardiovascular-derived scoring system may classify a clinically appropriate behaviour as lower MedD adherence. No tool included in this review was developed or validated specifically for breast cancer survivors, and no study reported formal validation of its scoring system in this population. This variation in measurement limits the synthesis of the MedD evidence in breast cancer survivorship and is more important than any individual outcome finding.

Across the 20 included studies, MedD adherence was examined against a broad but unevenly assessed range of outcomes: most frequently anthropometric measures (16 studies) and less often blood lipids (11 studies), glycaemic markers (12 studies), inflammatory markers (5 studies), QoL (4 studies), and mortality/recurrence (1 study) (Table 3). Interpretation of these findings should account for factors that may independently influence both MedD adherence and the outcomes assessed, including physical activity, menopausal status, endocrine therapy, socioeconomic status, and baseline metabolic risk; few included studies adjusted for these factors, limiting causal interpretation of the reported associations. Findings were somewhat more consistent for anthropometric outcomes, although significant between-group differences favouring the MedD intervention over a comparator were demonstrated in only a minority of controlled studies. Findings for blood lipids, glycaemic control, inflammatory markers, and QoL were mixed both within and across studies. Mortality and recurrence were assessed only by Kim et al. (2011) [45], which found no association between MedD adherence and breast-cancer-specific outcomes, highlighting a substantial evidence gap.

This is a notable gap given that MedD adherence is consistently associated with reduced cancer risk [28] and reduced all-cause and cardiovascular mortality [29] in general and mixed-cancer populations; there is limited evidence of independent replication specifically in breast cancer survivors. This is clinically relevant given that weight gain and adiposity after breast cancer diagnosis have been associated with poorer prognosis and treatment-related morbidity [8,9]. The relatively consistent improvements in anthropometric outcomes observed across the included studies may therefore be clinically meaningful, although whether these changes translate into improved recurrence or survival outcomes remains uncertain. Given the tool heterogeneity described above, together with variation in study design and follow-up, these findings are best interpreted as mapping the range and direction of outcomes examined to date, rather than as evidence for or against the effectiveness of MedD-based interventions in this population.

Several of the largest and most frequently reported studies in this review delivered MedD counselling as part of a broader lifestyle intervention that included structured exercise or other lifestyle advice [39,40,41,47,49,55,61]. In these studies, improvements in anthropometric and metabolic outcomes cannot be confidently attributed to MedD adherence independently of concurrent changes in physical activity.

Gnagnarella et al. (2026) [50] is a notable exception, including a diet-only arm alongside a combined diet-and-physical-activity arm, allowing some separation of dietary and activity-related effects for the weight loss outcome. This type of design was uncommon among the included studies. Future studies that isolate dietary intervention from exercise, could help clarify the independent contribution of MedD adherence to observed outcomes.

The broader relationship between diet and breast cancer outcomes has been investigated using approaches beyond named MedD-specific tools, including general diet-quality indices and –data-derived dietary patterns [64]. These studies were outside the scope of this review because our focus was specifically on MedD adherence instruments. However, differences between dietary assessment tools are evident even within individual studies. For example, Kim et al. (2011) [45] assessed diet quality in the same cohort using four different indices: aMED, the Alternate Healthy Eating Index, the Diet Quality Index-Revised, and the Recommended Food Score. The Recommended Food Score was linked to a borderline increased risk of breast cancer mortality overall, while aMED and the Diet Quality Index-Revised were linked to lower risk of death from other causes, mainly cardiovascular and respiratory, in physically inactive women only. Neither was associated with lower breast cancer mortality, highlighting that both the dietary tool and outcome examined can influence the observed relationship between diet and survival.

Several individual and treatment-related factors not systematically extracted in this review, including time since treatment completion, endocrine therapy use, and menopausal status, are recognised to influence both dietary behaviour and metabolic health in breast cancer survivors [65,66,67] and may potentially have contributed to the variability in adherence and outcomes observed across the included studies. Survivors in the acute post-treatment phase commonly report treatment-related barriers to healthy eating [68], while endocrine therapies such as tamoxifen and aromatase inhibitors are associated with weight gain and metabolic change [69]. These factors were not a specific focus of this review and were inconsistently reported across the included studies, limiting formal examination of their influence here; however, their potential relevance suggests they warrant consideration as effect modifiers in future research on MedD adherence in this population.

### 4.1. Limitations

Several methodological limitations must be acknowledged. The review protocol was registered retrospectively on the OSF following the initial search, rather than prospectively before literature screening commenced. Consequently, registration did not provide a prospective public record of the review methods; however, the final protocol, rationale for amendments, and review procedures are reported transparently.

Most studies relied on self-reported dietary data, introducing potential recall and reporting bias. Few studies incorporated objective biomarkers of adherence or explored the behavioural determinants of dietary change, both of which would strengthen validity and better inform intervention design. Study design also limited the strength of the evidence base. Many studies were underpowered and did not adjust for confounders such as physical activity, menopausal status, endocrine therapy, socioeconomic status, baseline metabolic risk or treatment type, while short intervention durations, such as two months in some trials, prevent evaluation of long-term adherence or sustainability.

A further limitation is the inconsistent definition, measurement, and reporting of adherence (Table 2), which limits comparability across studies and prevents robust synthesis of the findings. Ten different MedD scoring tools were used, incorporating 9–18 dietary components, with differing scoring approaches and adherence thresholds applied inconsistently across studies. This heterogeneity, combined with variation in study design and outcome reporting, means findings across studies cannot be directly compared or meta-analysed.

Finally, the predominance of European cohorts limits generalisability to non-Mediterranean populations, where food availability and cultural preferences differ.

### 4.2. Implications for Future Research and Clinical Practice

Future research should prioritise larger, longitudinal studies to better characterise the relationship between MedD adherence and health outcomes in women who have completed breast cancer treatment. Prospective, comparative studies examining MedD-based approaches alongside current nutrition guidance could clarify whether different dietary approaches offer distinct benefits and whether the evidence is sufficient to inform future guideline development. The use of objective dietary biomarkers of adherence (rather than self-report alone), and longer follow-up periods would also be beneficial. Addressing the measurement inconsistencies identified in this review (Table 2) will be essential for meaningful comparison and future meta-analysis. Researchers should use validated, standardised MedD scoring tools with clearly defined, prespecified adherence thresholds and report these consistently across studies. There is also a case for evaluating the MedD specifically in breast cancer survivors, rather than relying solely on general cancer-prevention guidance such as WCRF recommendations. As noted earlier, the MedD’s endorsement for menopause symptom management [17] is particularly relevant given that many breast cancer survivors experience treatment-induced or accelerated menopause as a consequence of chemotherapy, ovarian suppression, or endocrine therapy [12,13]. This is an aspect of survivorship that may not be captured by generic cancer-prevention dietary guidance. This potential relevance is reflected in findings from White et al. (2025) [57], who reported a significant reduction in menopausal symptom severity alongside improved MedD adherence following a 12-week intervention. These findings suggest that the potential role of the MedD in breast cancer survivorship extends beyond cancer prevention alone. Further research is needed to determine whether the MedD can help address the specific symptoms and metabolic changes experienced by breast cancer survivors.

The choice of scoring tool also has practical implications. Fixed portion-based cut-offs (e.g., MEDAS-14) are simple and well validated but may not reflect population-specific dietary patterns, potentially misclassifying adherence in non-Mediterranean groups. Population-relative tools (e.g., OMDS) require a reference population and are less suitable for individual clinical use. Standard MEDAS-14 and PREDIMED give a favourable score for alcohol, which may be inappropriate for breast cancer survivors given the established cancer risk associated with alcohol. As discussed previously, alcohol-excluded versions (e.g., modified 13-item MEDAS) may be more appropriate for breast cancer survivors. Tools with a wider score range (e.g., QueMD, MDS) may also be better suited to tracking gradual dietary change over time rather than simply classifying adherence at one point.

If MedD scoring tools are to be applied in the Irish context, they should be adapted to reflect locally available and culturally relevant foods. For example, alcohol should be excluded in line with WCRF guidelines, and dairy should be included in line with national recommendations to address osteoporosis risk after breast cancer treatment [12,70]. Processed and red meats should be clearly distinguished, and culturally uncommon foods such as sofrito should be replaced with locally appropriate alternatives. Portion sizes should be specified as objectively as possible, using familiar measurements, to reduce variability in responses. Given the lack of specificity in current cancer survivorship guidelines relating to tumour-specific considerations—such as the impact of iatrogenic menopause on bone health, body composition, cardiovascular risk, sarcopenic obesity, and cognitive outcomes—the MedD may offer a useful nutritional framework to support these outcomes, reduce chronic disease risk, and potentially influence long-term survivorship.

However, before adoption in research or clinical practice, MedD scoring tools should be fully validated in the Irish context and among women who have survived breast cancer. Additionally, future studies could explore outcomes not yet assessed, such as bone health, and should include a clear rationale for the choice of scoring tool to ensure the appropriateness of the methodology for women after breast cancer treatment.

## 5. Conclusions

This scoping review identified a growing body of research examining MedD adherence among breast cancer survivors. While several interventions were associated with short-term improvements in adherence and metabolic health markers, methodological variability and small study sizes limit definitive conclusions on long-term clinical outcomes. In particular, evidence relating MedD adherence to recurrence and survival remains extremely limited, drawn from a single cohort study, and should be prioritised in future research. There is also a case for evaluating the MedD specifically within breast cancer survivorship—rather than through generic cancer-prevention frameworks alone—given emerging evidence linking MedD adherence to improvements in menopausal symptoms, a symptom burden distinct to this population. Standardising MedD assessment tools, ensuring cultural adaptation, and conducting well-powered longitudinal studies will be essential to establish the potential role of MedD-based approaches in breast cancer survivorship care.

## Figures and Tables

**Table 3:** Evidence-gap matrix: clinical, metabolic, and quality-of-life outcome domains assessed by study.

Study	Design	Mortality	Anthropometric	Blood Lipids	Glycaemic	Inflammatory	Quality of Life
Kim 2011 [45]	Cohort	^M^	—	—	—	—	—
Finocchiaro 2016 [59]	Single-arm	—	↓ ᵂ	—	—	—	—
Braakhuis 2017 [7]	RCT	—	↓ ᴮ	^M^	↔	—	↔
Zuniga 2019 [60]	RCT	—	—	—	—	—	—
Magro 2019 [47]	RCT	—	↓ ᵂ (1 arm)	—	—	—	—
Cho 2020 ^b^ [42]	RCT	—	↓ ᵂ (all)	↓ ᵂ (2 arms)	↓ ᵂ (2 arms)	—	↑ ᵂ
Ruiz-Vozmediano 2020 [55]	RCT	—	↓ ᴮ	^M^	↔	—	↑ ᴮ
Kwon 2020 ^b^ [43]	Single-arm	—	↓ ᵂ	—	↓ ᵂ	—	—
Pellegrini 2020 [58]	RCT	—	↓ ᵂ (both)	↔	^M^	↔	—
Natalucci 2023 ^a^ [40]	Single-arm	—	^M^	^M^	↓ ᵂ	↓ ᵂ	—
Negrati 2021 [48]	Cohort	—	↔	↔	↔	—	—
Alvarez-Bustos 2021 [56]	Cross-sectional	—	—	—	—	—	↔
Acito 2023 [46]	Cohort	—	—	—	—	—	—
Garcia-Unciti 2023 [61]	RCT	—	↓ ᴮ	—	—	—	—
Donati Zeppa 2023 ^a^ [39]	Single-arm	—	↔	↓ ᵂ	^M^	↔	—
White 2025 [57]	Single-arm	—	↓ ᵂ	—	—	—	—
Ruiz-Molina 2025 [49]	RCT	—	↓ ᴮ	↔	↔	↔	—
Annibalini 2026 ^a^ [41]	RCT	—	↓ ᵂ	^M^	^M^	—	—
Choi 2026 ^b^ [44]	RCT	—	↓ ᵂ	^M^	^M^	—	—
Gnagnarella 2026 ^c^ [50]	RCT	—	↓ ᴮ	↔	↓ ᵂ	↔	—

**↓/↑** Statistically significant decrease/increase reported. ᴮ Significance was between-group (i.e., RCT comparison vs. a control/comparator arm). ᵂ Significance was within-group only—either no significant between-group difference was found despite an RCT/multi-arm design, or the study design (single-arm, cohort) does not permit a between-group comparison. ↔ **The** domain was assessed; no statistically significant change was reported. ^M^ Mixed findings—direction or significance differs across sub-outcomes, study arms, or subgroups. — Domain was not assessed. **^a^** MoviS trial (NCT04818359). **^b^** Korean trial (NCT03581630). **^c^** InForma trial (NCT02622711).

## Data Availability

Data supporting the findings of this review are available from the corresponding author upon reasonable request.

## Supplementary Materials

The following supporting information can be downloaded at https://www.mdpi.com/article/10.3390/cancers18193185/s1, Table S1: Search Strategy; Table S2: Specific Components of Mediterranean Dietary Score and Operationalisation of Recommendations.
