# Supplementary material for: Mediterranean Diet Adherence Tools in Post-Treatment Breast Cancer Survivors: A Scoping Review of Measurement Approaches and Associated Outcomes
Source: Cancers (Basel). 2026 Oct 2;18(19):3185. doi: 10.3390/cancers18193185 (PMC13664086; doi:10.3390/cancers18193185)
Supplement: Supplementary file 1 [file cancers-18-03185-s001.zip › Supplementary Table S1.pdf]

Table S1. Search Strategy.

Database	Search Strategy
PubMed	("Diet, Mediterranean"[MeSH] OR "Mediterranean Diet"[tiab] OR "Med Diet"[tiab] OR "MedDiet"[tiab] OR "Mediterranean diet adherence"[tiab] OR "Mediterranean Diet score*"[tiab] OR "Mediterranean Diet scoring"[tiab] OR "mediterranean dietary pattern"[tiab] OR "anti-inflammatory diet"[tiab] OR "anti inflammatory diet"[tiab]) AND ("Breast Neoplasms"[MeSH] OR "breast cancer"[tiab] OR "breast neoplasm*"[tiab] OR "breast tumor*"[tiab] OR "breast tumour*"[tiab] OR "breast carcinoma*"[tiab] OR "breast adenocarcinoma*"[tiab] OR "mammary cancer"[tiab] OR "breast malignancy"[tiab]) AND ("Cancer Survivors"[MeSH] OR "survivor*"[tiab] OR "survivorship"[tiab] OR "post treatment"[tiab] OR "post-treatment"[tiab] OR "after treatment"[tiab] OR "post therapy"[tiab] OR "finished treatment"[tiab] OR "after completion of treatment"[tiab])
Web of Science	TS=("Mediterranean diet*" OR "med diet" OR "meddiet" OR "Mediterranean dietary pattern*" OR "Mediterranean diet adherence" OR "Mediterranean diet score*" OR "Mediterranean diet scoring" OR "anti-inflammatory diet*" OR "anti inflammatory diet*") AND TS=("breast cancer*" OR "breast neoplasm*" OR "breast carcinoma*" OR "breast adenocarcinoma*" OR "breast tumour*" OR "breast tumor*" OR "mammary cancer" OR "breast malignancy") AND TS=("survivor*" OR "survivorship" OR "cancer survivorship" OR "post-treatment" OR "post treatment" OR "after treatment" OR "after completion of treatment" OR "finished treatment" OR "post therapy")
Embase Elsevier	('mediterranean diet'/exp OR 'mediterranean diet*':ti,ab OR 'med diet':ti,ab OR 'meddiet':ti,ab OR 'med-diet':ti,ab OR 'mediterranean-style diet':ti,ab OR 'mediterranean dietary pattern*':ti,ab OR 'mediterranean diet adherence':ti,ab OR 'mediterranean diet score*':ti,ab OR 'mediterranean diet scoring':ti,ab OR 'anti-inflammatory diet*':ti,ab OR 'anti inflammatory diet*':ti,ab) AND ('breast neoplasm'/exp OR 'breast cancer*':ti,ab OR 'breast neoplasm*':ti,ab OR 'breast carcinoma*':ti,ab OR 'breast adenocarcinoma*':ti,ab OR 'breast tumour*':ti,ab OR 'breast tumor*':ti,ab OR 'mammary cancer':ti,ab OR 'breast malignancy':ti,ab) AND ('cancer survivor'/exp OR 'survivor*':ti,ab OR 'survivorship':ti,ab OR 'cancer survivorship':ti,ab OR 'post-treatment':ti,ab OR 'post treatment':ti,ab OR 'after treatment':ti,ab OR 'after completion of treatment':ti,ab OR 'finished treatment':ti,ab OR 'post therapy':ti,ab OR 'beyond treatment':ti,ab)
Scopus	TITLE-ABS-KEY("mediterranean diet*" OR "med diet" OR "meddiet" OR "mediterranean style diet" OR "mediterranean dietary pattern*" OR "mediterranean diet adherence" OR "mediterranean diet scoring" OR "anti-inflammatory diet*" OR "anti inflammatory diet*") AND TITLE-ABS-KEY("breast cancer*" OR "breast neoplasm*" OR "breast carcinoma*" OR "breast adenocarcinoma*" OR "mammary cancer" OR "breast tumour*" OR "breast tumor*" OR "breast malignancy") AND TITLE-ABS-KEY("survivor*" OR "cancer survivorship" OR "survivorship" OR "post-treatment" OR "post treatment" OR "after treatment" OR "after completion of treatment" OR "finished treatment" OR "post therapy")
Cinahl	(MH "Mediterranean Diet" OR TX ("Mediterranean diet*" OR "Med diet" OR "MedDiet" OR "Med-diet" OR "Mediterranean-style diet" OR "Mediterranean dietary pattern*" OR "Mediterranean diet adherence" OR "Mediterranean diet score*" OR "Mediterranean diet scoring" OR "anti-inflammatory diet*" OR "anti inflammatory diet*")) AND (MH "Breast Neoplasms+" OR TX ("breast cancer*" OR "breast neoplasm*" OR "breast carcinoma*" OR

	"breast adenocarcinoma*" OR "breast tumour*" OR "breast tumor*" OR "mammary cancer" OR "breast malignancy")) AND (MH "Cancer Survivors" OR TX ("survivor*" OR "survivorship" OR "cancer survivorship" OR "post-treatment" OR "post treatment" OR "after treatment" OR "after completion of treatment" OR "finished treatment" OR "post therapy" OR "beyond treatment"))
