# Supplementary material for: Mediterranean Diet Adherence Tools in Post-Treatment Breast Cancer Survivors: A Scoping Review of Measurement Approaches and Associated Outcomes
Source: Cancers (Basel). 2026 Oct 2;18(19):3185. doi: 10.3390/cancers18193185 (PMC13664086; doi:10.3390/cancers18193185)
Supplement: Supplementary file 1 [file cancers-18-03185-s001.zip › Supplementary Table S2.pdf]

Table S2. Specific Components of Mediterranean Dietary Score and Operationalisation of Recommendations.

Mediterranean Dietary Component	**Mediterranean Dietary Scores and Operationalisation of Recommendations**																			
	**Cardioprotective Mediterranean Diet Score** *Martinez-Gonazales et al. 2004*		**MEDAS 14 ^** *Schnoder et al. 2011*		**aMED** *Kim et al. 2011*		**Mediterranean Diet Score** *Demetriou et al. 2012*		**MEDI-LITE** *Sofi et al. 2013*		**Italian Mediterranean Index ‡** *Agnoli et al. 2013*		**PREDIMED-PLUS Energy Restricted ~** *Martinez-Gonazales et al. 2019*		**Modified 13-item MEDAS †** *Cho et al. 2020; Kwon et al. 2020; Choi et al. 2026*		**QueMD** *Gnagnarella et al., 2026*		**Original Mediterranean Diet Score (OMDS) #** *Trichopoulou et al. 2003*	
Vegetables	✓	1 point = ≥ 1 servings/day<br><br>Additional point if ≥ 1 serving/day of vegetables *and* fruit	✓	1 point = ≥ 2 servings/day	✓	1 point if > median intake	✓	1 point if ≥ 1 serving/day<br><br>Additional point if ≥ 1 serving/day of vegetables *and* fruit	✓	0 = <1 portion/day<br>1 = 1-2.5 portions/day<br>2 = >2.5 portions/day	✓	1 point if > third tertile of distribution	✓	1 point = ≥ 2 servings/day (≥1 portion raw or in a salad)	✓	1 point = ≥ 2 servings/day	✓	1 point = ≥ 2 servings/day	✓	1 point if intake above sex-specific median
Legumes / Pulses	✓	1 point = ≥ 2 servings/week	✓	1 point = ≥ 3 servings/week	✓	1 point if > median intake	✓	1 point if ≥ 2 servings/week	✓	0 = <1 portion/week<br>1 = 1-2 portions/week<br>2 = >2 portions/week	✓	1 point if > third tertile of distribution	✓	1 point = ≥ 3 servings/week	✓	1 point = ≥3 servings/week	✓	1 point = ≥ 2 servings/week	✓	1 point if intake above sex-specific median
Fruits	✓	1 point = ≥ 1 servings/day<br><br>Additional point if ≥ 1 serving/day of vegetables *and* fruit	✓	1 point = ≥ 3 pieces/day	✓	1 point if > median intake	✓	1 point if ≥ 1 serving/day<br><br>Additional point if ≥ 1 serving/day of vegetables *and* fruit	✓	0 = <1 portion/day<br>1 = 1-1.5 portions/day<br>2 = >2 portions/day	✓	1 point if > third tertile of distribution	✓	1 point = ≥ 3 servings /day	✓	1 point = ≥3 servings/day	✓	1 point = ≥ 2 servings/day	✓	1 point if intake above sex-specific median (nuts included in this component)
Wholegrains / Cereals	✓	1 point = white bread <1 serving per day and rice <1 servings			✓	1 point if > median intake (only wholegrains included)	✓	1 point if 1-3 serving/day	✓	0 = <1 portion/day<br>1 = 1-1.5 portions/day	✓	1 point if intake of pasta is > third tertile	✓	1 point = ≤1 serving of white bread per day	✓	≤1 serving white bread/day	✓	1 point = ≥ 1 servings/day	✓	1 point if intake above sex-specific median (all cereal

		per week *or* wholegrain bread >5 servings per week								2 = >1.5 portions/day		of distribution		1 point = ≥5 times per week of wholegrain cereals and pasta<br><br>1 point = <3 times per week of non-whole grain pasta or white rice						products, not restricted to wholegrain)
Dried Fruits																	✓	1 point = ≥ 2 servings/week		
Potatoes											✓	1 point in intake is < 1st tertile of distribution								
Fish / Seafood	✓	1 point = ≥ 3 servings/week	✓	1 point = ≥ 3 servings/week	✓	1 point if > median intake	✓	1 point if ≥ 3 servings/week	✓	0 = <1 portion/week<br>1 = 1-2.5 portions/week<br>2 = >2.5 portions/day	✓	1 point if > third tertile of distribution	✓	1 point = ≥ 3 servings/week	✓	≥3 servings/week	✓	1 point = ≥ 2 servings/week	✓	1 point if intake above sex-specific median
Poultry / Lean Meats			✓	1 point if white meat is preferred over red meat									✓	1 point if white meat is preferred over red meat	✓	1 point if white meat preferred over red meat	✓			
Red Meat	✓	1 point = < 1 serving/day *	✓	1 point = < 1 serving/day *	✓	1 point if < median intake *	✓	1 point if < 1 serving/day +	✓	0 = >1.5 portion/day<br>1 = 1-1.5 portions/day<br>2 = <1 portion/day +	✓	1 point in intake is < 1st tertile of distribution	✓	1 point = < 1 serving/day *	✓	1 point = <1 serving/day	✓	1 point = ≤1 to 3 times/ week	✓	1 point if intake below sex-specific median *
Processed Meats													✓	1 point = < 1 serving/day *	✓	1 point = <1 serving/day			✓	1 point if intake below sex-specific median *

Dairy				✓	1 point if < median intake			✓	0 = <1.5 portion/day<br>1 = 1-1.5 portions/day<br>2 = <1 portion/day					-				✓	1 point if intake below sex-specific median
Butter / Cream / Margarine		✓	1 point = < 1 serving/day of animal fat							✓	1 point in intake of butter is < 1st tertile of distribution	✓	1 point = < 1 serving/day	✓	1 point = <1 serving/day				
Olive Oil		✓	1 point if used as primary cooking fat<br>1 point if ≥ 4 tbsp/day	✓	1 point if ratio of monounsaturated fat to saturated fat is > median ration	✓	1 point if ≥ 1 spoon/day	✓	0 = occasional use<br>1 = frequent use<br>2 = regular use	✓	1 point if > third tertile of distribution	✓	1 point if extra virgin olive oil is primary oil used for cooking, spreads and salad dressings	✓	1 point if used as primary cooking fat; 1 point if ≥4 tbsp/day	✓	1 point if ≥3 tbsp/day		
Nuts		✓	1 point = ≥ 3 servings/week									✓	1 point = ≥ 3 servings/week	✓	1 point = ≥3 servings/week				
Tomato / Onion / Leek Sauce		✓	1 point if ≥ 2 servings/week									✓	1 point if ≥ 2 servings/week	✓	1 point if ≥2 servings/week				
Dessert / Sweets															-				
Commercial Cakes		✓	1 point if < 2 per week									✓	1 point if < 3 per week	✓	1 point if <3 per week				
Fizzy Drinks / Sugar Sweetened Soft Drinks		✓	1 point if < 1 cup/day (100 ml)							✓	1 point in intake is < 1st tertile of distribution	✓	1 point = <1 sugary beverages or sugar-sweetened fruit juices per week	✓	1 point = <1 sugary beverage/week				

														1 point if avoid adding sugar to beverages						
Alcohol	✓	1 point = ≥ 1 glass per day	✓	1 point if ≥ 7 servings/week of red wine	✓	1 point = 5 - 15g/day	✓	1 point if zero consumption	✓	0 = >2 AU/day<br>1 = <1 AU/day<br>2 = 1-2 AU/day	✓	1 point = ≤12 g/day<br>0 point = <12g/day or abstainers	✓	1 point = 1-2 glasses of wine per day	*(not scored — item removed)*		✓	1 point = ≤1 glass of wine per day	✓	1 point if moderate consumption (men: 10–50 g/day; women: 5–25 g/day)
Maximum Score	9		14		9		9		18		11		17		13		9		9	

* Red meat and processed meat included together.

+ All meat products included in score. † Cho et al. 2020 (parent trial), Kwon et al. 2020 (nested sub-study of Group A participants from the same trial), and Choi et al. 2026 (secondary/exploratory analysis from the same trial) all applied the same modified 13-item MEDAS instrument (alcohol item removed, 0–13 scale). Kwon et al. (2020) used a Korean-adapted version (K-MEDAS).

^ The following studies included in this scoping review used the MEDAS 14 scoring tool to assess MedD adherence: Alvarez-Bustos et al. 2021; Braakhuis et al. 2017; Natalucci et al. 2023; Ruiz-Vozmediano et al. 2020; Zeppa et al. 2023; White et al. 2025; and Annibalini et al. 2026.

‡ The following studies included in this scoping review used the Italian Mediterranean Index to assess MedD adherence: Pellegrini et al. 2020; Finocchiaro et al. 2016.

~ The following studies included in this scoping review used the PREDIMED-PLUS Energy-Restricted MedDiet: Ruiz-Molina et al. 2025; Acito et al. 2023.

Ratio of monounsaturated to saturated fat intake used as the fat-quality criterion in OMDS, rather than olive oil intake specifically.
